# Volatiles from cotton aphid (*Aphis gossypii*) infested plants attract the natural enemy *Hippodamia variegata*

**DOI:** 10.3389/fpls.2023.1326630

**Published:** 2023-12-20

**Authors:** Chaoqun Yi, Dong Teng, Jiaoxin Xie, Haoyu Tang, Danyang Zhao, Xiaoxu Liu, Tinghui Liu, Wei Ding, Adel Khashaveh, Yongjun Zhang

**Affiliations:** ^1^ State Key Laboratory for Biology of Plant Diseases and Insect Pests, Institute of Plant Protection, Chinese Academy of Agricultural Sciences, Beijing, China; ^2^ Key Laboratory of Entomology and Pest Control Engineering, College of Plant Protection, Southwest University, Chongqing, China; ^3^ Key Laboratory of Integrated Management of Crop Diseases and Pests (Ministry of Education), College of Plant Protection, Nanjing Agricultural University, Nanjing, China; ^4^ College of Animal Science, Shanxi Agricultural University, Jinzhong, China; ^5^ School of Resources and Environment, Henan Institute of Science and Technology, Xinxiang, China; ^6^ National Key Laboratory of Green Pesticide, College of Plant Protection, South China Agricultural University, Guangzhou, China; ^7^ College of Plant Protection, Hebei Agricultural University, Baoding, China

**Keywords:** cotton aphid, cotton, HIPV, natural enemy, attraction

## Abstract

The *Aphis gossypii* is a major threat of cotton worldwide due to its short life cycle and rapid reproduction. Chemical control is the primary method used to manage the cotton aphid, which has significant environmental impacts. Therefore, prioritizing eco-friendly alternatives is essential for managing the cotton aphid. The ladybird, *Hippodamia variegata*, is a predominant predator of the cotton aphid. Its performance in cotton plantation is directly linked to chemical communication, where volatile compounds emitted from aphid-infested plants play important roles in successful predation. Here, we comprehensively studied the chemical interaction between the pest, natural enemy and host plants by analyzing the volatile profiles of aphid-infested cotton plants using gas chromatography-mass spectrometry (GC-MS). We then utilized the identified volatile compounds in electrophysiological recording (EAG) and behavioral assays. Through behavioral tests, we initially demonstrated the clear preference of both larvae and adults of *H. variegata* for aphid-infested plants. Subsequently, 13 compounds, namely α-pinene, *cis*-3-hexenyl acetate, 4-ethyl-1-octyn-3-ol, β-ocimene, dodecane, E-β-farnesene, decanal, methyl salicylate, β-caryophyllene, α-humulene, farnesol, DMNT, and TMTT were identified from aphid-infested plants. All these compounds were electrophysiologically active and induced detectable EAG responses in larvae and adults. Y-tube olfactometer assays indicated that, with few exceptions for larvae, all identified chemicals were attractive to *H. variegata*, particularly at the highest tested concentration (100 mg/ml). The outcomes of this study establish a practical foundation for developing attractants for *H. variegata* and open avenues for potential advancements in aphid management strategies by understanding the details of chemical communication at a tritrophic level.

## Introduction

*Aphis gossypii* (Hemiptera: Aphididae), commonly known as the cotton aphid, is one of the major threats to cotton fields globally. Nymphs and adults cause substantial damage to the stems and leaves of cotton plants using their piercing and sucking mouthparts. This feeding behavior leads to significant nutritional loss in cotton, affecting its overall productivity. Furthermore, the cotton aphid excretes honeydew and transmits viral diseases. The short life cycle and rapid reproduction of aphids contribute to the continuous presence of multiple generations within a single year, resulting in a large population. The combined impact of these factors severely compromises both the yield and quality of cotton, making effective control measures crucial for cotton growers (Wang et al., 2016; Zhang Z. et al., 2020).

Currently, chemical control is the primary method used to manage cotton aphids (Kerns and Stewart, 2000), however, this approach has significant environmental impacts. Excessive use of insecticides can lead to increased aphid resistance (Ma et al., 2019; Zhang H. et al., 2020), higher pesticide residues in soil (Hua et al., 2023), and harm to natural enemies of insects. The decline of natural enemies can cause a surge in pest populations and disrupt the ecological balance of farmland (Das and Rahman, 2023; Dunn et al., 2023; McClure et al., 2023; Seni, 2023). Hence, it is imperative to prioritize sustainable and environmentally friendly alternatives to chemical control in order to efficiently manage cotton aphids.

There are numerous natural enemies of insect pests in nature, such as parasitic wasps (Daniel et al., 2023), ladybirds, and lacewings (Wang et al., 2022; González-Ruiz et al., 2023). Additionally, arthropods like spiders and mites are capable of preying on a diverse range of pests (Vervaet et al., 2022; Fidelis et al., 2023). Employing natural enemies for pest control provides multiple advantages over insecticides, including reduced environmental pollution caused by chemical agents. Natural enemies not only effectively control the population of pests, but also maintain the population of other insects in the field and preserve the ecological balance (Karamaouna et al., 2021; Leung et al., 2022; Souza et al., 2023).

As a member of the ladybird family (Coleoptera: Coccinellidae), both adults and larvae of *Hippodamia variegata* (Goeze) possess the ability to prey on a large number of cotton aphids (Franzmann, 2002; Pervez et al., 2018; Nordey et al., 2021), making them a valuable natural enemy in Xinjiang, China, where cotton fields are prevalent (Dou et al., 2023). Utilizing *H. variegata* for cotton aphid control not only helps mitigate aphid resistance, but also aligns with the principles of environmentally friendly pest management, offering a promising approach for integrated pest management (IPM) (Baker et al., 2020; Jack and Ellis, 2021; Overton et al., 2021).

The chemical communication system in insects is highly complex and plays a vital role in various insect behaviors, including foraging, mating, host location, and habitat selection (Schiestl, 2010; Webster and Carde, 2017; Adams et al., 2020; Ebrahim et al., 2023). To successfully engage in predation and complete the task, sensitivity to volatile compounds is crucial for natural enemy insects. They detect volatile compounds emitted by plants, locate the plants, and prey on the pests infesting them. The volatile compounds released by plants, primarily during the flowering stage, assist natural enemies in locating supplementary food sources such as pollen and nectar, which are essential for their reproduction and growth (Schuldiner-Harpaz and Coll, 2017; Zhao et al., 2020). More importantly, natural enemy insects possess the remarkable ability to directly detect and track the intricate chemical signatures emitted by their potential hosts. Combinatory perception of plant/host-derived semiochemicals from a complex environment facilities effective host location and subsequent parasitism or predation (Leroy et al., 2011; Sablon et al., 2012; Monticelli et al., 2020; Gallon and Smilanich, 2023).

It has been reported in numerous studies that when pests damage plants, the plants release specific volatile components known as herbivore-induced plant volatiles (HIPVs) (Heil, 2008; Dicke and Baldwin, 2010; Turlings and Erb, 2018; Erb et al., 2021). HIPVs are a diverse group of volatile organic compounds (VOCs) that released from leaves, flowers, and fruits into the atmosphere and from roots into the soil. They are synthesized from a variety of precursors, including fatty acids, amino acids, and terpenes (Dicke and Baldwin, 2010; Ali et al., 2023). They play important roles in plant indirect defense by attracting natural enemies of herbivores in various ways. First, they can act as cues that indicate the presence of a food source. Second, HIPVs can provide information about the type of herbivore that is attacking the plant. This allows natural enemies to specialize on particular herbivore species. Third, HIPVs can disorient herbivores and make it difficult for them to escape from natural enemies (Dicke and Baldwin, 2010; Hare, 2011; Erb et al., 2021; Manzano et al., 2022; Khallaf et al., 2023). HIPVs also have a direct impact on herbivores and can interfere with their feeding or reproduction (Valle et al., 2023; Yang et al., 2023). In addition, HIPVs can also prime plants for further defense. When a plant is exposed to HIPVs from a neighboring plant that is being attacked by herbivores, the plant will begin to produce its own defenses in preparation for a possible attack. This priming process can help to reduce the amount of damage that the plant sustains if it is attacked by herbivores (Turlings and Erb, 2018; Ghosh et al., 2023).

In this study, our hypothesis revolves around the potential alteration in cotton plant volatile profiles following infestation by cotton aphid, consequently influencing the emission of specific volatiles. We speculate that these volatiles play crucial roles in modulating the predatory behavior of *H. variegata*. To substantiate this hypothesis and delve deeper into the chemical communication between *H. variegata*, cotton plants, and cotton aphid, our research was performed in a structured approach as follows: 1) a dual-choice behavioral assay was conducted to assess the attraction of both *H. variegata* adults and larvae to aphid-infested and uninfested cotton plants, 2) volatile collection and subsequent analysis via gas chromatography-mass spectrometry (GC-MS) were carried out to determine the volatile profiles of aphid-infested and uninfested cotton plants, 3) electroantennogram (EAG) and Y-tube olfactometer experiments were employed to evaluate the electrophysiological and behavioral responses of *H. variegata* adults and larvae to the identified volatiles, respectively. We aimed to elucidate how changes in volatile emissions might influence the predatory behavior of *H. variegata*. Ultimately, the findings from this study are anticipated to provide valuable insights into optimizing the utilization of the natural enemy, *H. variegata*, for effective biological control of cotton aphids (*A. gossypii*). The incorporation of specific volatile compounds into control strategies presents a promising and sustainable approach, potentially reducing reliance on chemical insecticides and promoting IPM practices.

## Materials and methods

### Insects and plants

The initial populations of *A. gossypii*, *H. variegata*, and the cotton seeds (*Gossypium hirsutum* L., cv. CCRI49) were obtained from the National Plant Protection Scientific Observation and Experiment Station, Langfang, China. The cotton aphid was reared in 30 cm × 30 cm × 50 cm net cages on cotton seedlings with 5-6 true leaves. The *H. variegata* were reared within the same size cages containing cotton seedlings infested with cotton aphids. Approximately 100 ladybirds (1:1 ratio for male and female) were released into the cages and the eggs were collected daily. New aphid sources were provided every other day. Third instar larvae and 2–5-day old male and female adults (unmated) were used for the behavioral assays and EAG recording. The adults were kept individually within 10 ml glass jars covered with wet cotton balls and were starved for 12 h prior to the experiments. The cotton seeds were planted in nursery pots (height: 15 cm, diameter: 15 cm), and were used in the experiments when they reached the 6-7 true leaf stage. All insect colonies and cotton plants were maintained in artificial climate chambers under controlled conditions (temperature: 27 ± 2°C; relative humidity: 65 ± 5%; photoperiod: 16 hours light/8 hours dark).

### HIPV induction

To induce HIPV emission, each cotton plant was randomly infested with approximately 300 cotton aphids, and the plant were kept in the artificial climate chamber for at least 48 hours. After this period, the aphids where carefully removed from the cotton plants using a fine brush without causing any mechanical damage. Nevertheless, the honeydew residues remained after aphid removal, potentially leading to the presence of additional compounds apart from HIPVs. The infested plants were immediately used for behavioral assays. The healthy cotton plants were used as controls.

### Dual-choice behavioral assay

**Figure 1:**
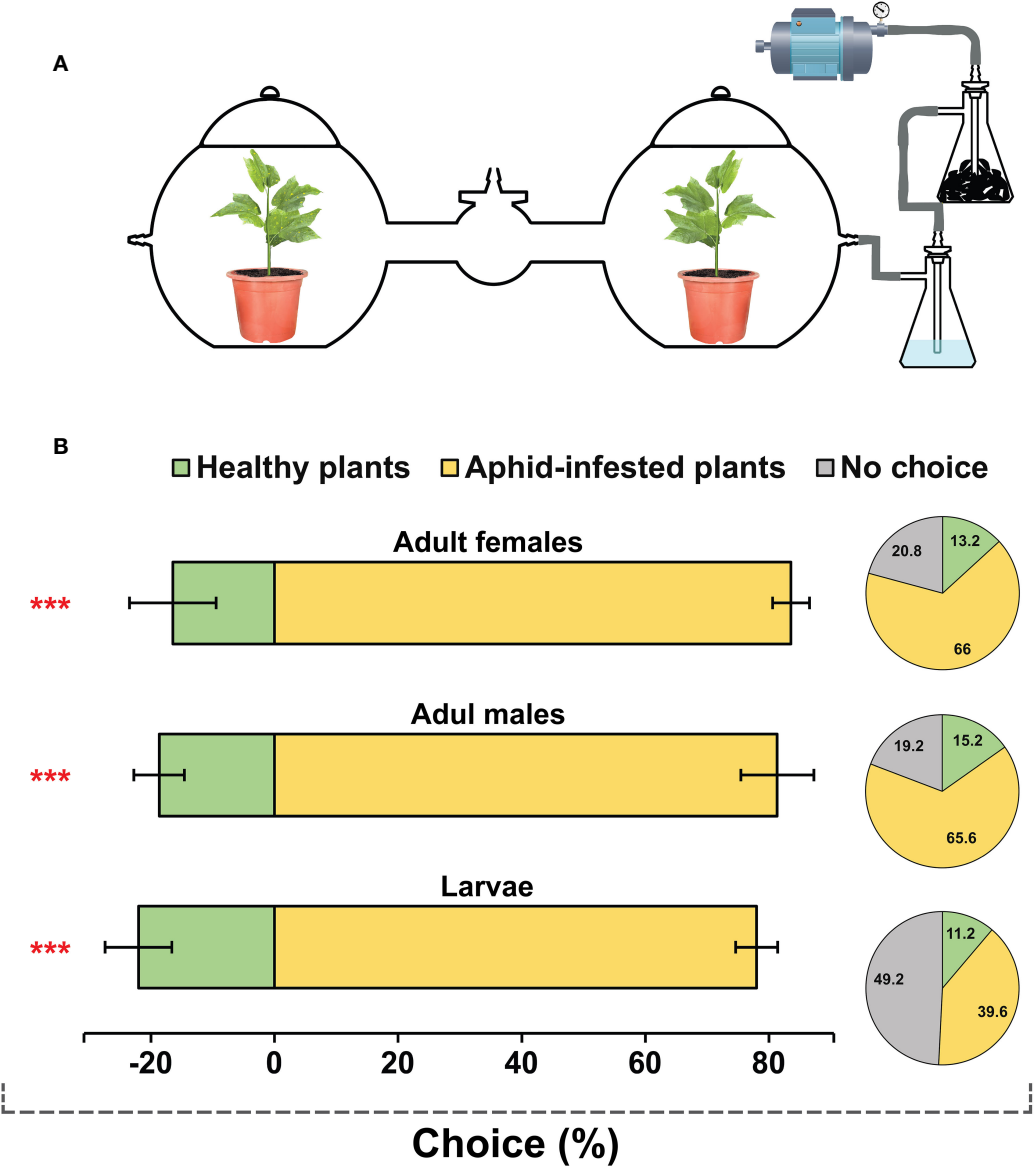
Behavioral responses of *Hippodamia variegata* to healthy plants and plants damaged by cotton aphid. **(A)** Schematic diagram of the dual-choice behavioral assay setup. **(B)** Bar charts demonstrate behavioral tendencies of third instar larvae, adult males and adult females towards healthy and damaged plants (proportion of insects that made choices). The data are shown as mean ± SEM. *** demonstrates significant differences (*P* < 0.001) analyzed by a chi-squared test. Pie charts illustrate the overall proportion of all tested insects, including those that made no choices and remained unresponsive.

To perform the behavioral assay, a dual-choice olfactometer was utilized in this study (**Figure 1A**). The entire apparatus was made of transparent glass and consisted of two flat-bottom spherical chambers (height: 40 cm, width: 40 cm). Each chamber was equipped with an air inlet on the side and a lid on the top, allowing for the delivery of clean air to each chamber and preventing any potential air leakage, respectively. The chambers were connected by an arm (length: 40 cm, internal diameter: 10 cm) equipped with an insect release chamber (height: 15 cm, width: 15 cm) at the midpoint. The aphid-infested or uninfested cotton plants were placed in the side chambers, and a constant charcoal-filtered humid air flow was delivered to the side chambers at the rate of 0.3 L/min and exhausted from the release chamber. The *H. variegata* individuals (fifty males, females, or larvae) were introduced simultaneously into the insect release chamber and allowed to make choices. The number of insects entering each chamber was counted every 30 minutes for a period of 6 hours, and the cumulative count was subjected to subsequent data analysis. Insects remaining in the release chamber or lateral arms were considered to have no response. The experiments were carried out with five replications under conditions similar to colony maintenance.

### Volatile collection

The volatiles emitted from healthy cotton plants or cotton aphid-infested plants were collected using headspace-solid phase microextraction (HS-SPME) following previously described methods (Avellaneda et al., 2021; Zhang et al., 2022). In brief, the pot and the soil surface surrounding the cotton stem were carefully wrapped with aluminum foil. The cotton plant was then enclosed in a cylindrical plastic container (height: 30 cm, diameter: same as pot) to create a controlled headspace environment. The interface between the pot and container was carefully sealed with food grade stretch film to prevent any air leakage. An empty container was included as a negative control. To trap the headspace volatiles, a SPME fiber (57310-U, Merck company) was inserted into the container and left for 8 hours. After the collection period, the fiber was removed and immediately used for GC-MS analysis. Each treatment was conducted with five replicates.

### Volatile analysis

The collected samples were identified using a Shimadzu GC-MS QP2020 system. The column used was Rxi-5Sil (Agilent Technologies, CA, USA) with a length of 30 m, a film thickness of 0.25 μm, and an inner diameter of 0.25 mm. Purified helium was used as the carrier gas with a flow rate of 1.0 ml/min. The inlet temperature, pressure, and septum purge flow were kept at 250°C, 45.8 kPa, and 3.0 ml/min, respectively. The GC was operated under the following conditions: the initial oven temperature was set to 30°C, which was then increased to 150°C at a rate of 5°C/min, held for 5 min, followed by a further increase to 250°C at a rate of 6°C/min. Finally, the temperature was raised to 280°C at a rate of 8°C/min and held for 5 minutes. For MS, the transfer line, the source, and the quad were maintained at temperatures of 280°C, 230°C, and 150°C, respectively. Mass spectra were taken in electron ionization (EI) mode at 70 eV, covering a range from 35 m/z to 500 m/z, with a scanning rate of 1666 amu/s. The compounds were identified by comparing their retention times and mass spectra with those available in the NIST17 library (National Institute of Science and Technology software, USA). The external standard approach was employed to quantify the relative content of each identified volatile within the collected samples, following the previous protocol (Rajana et al., 2019). Briefly, the standard solutions (1, 10, 50, 100, and 1000 ng/μl) of each compound were prepared in n-hexane (**Supplementary Table S1**). Each standard solution was then examined to evaluate the linearity and range. The quantity of each chemical was measured based on the peak area. The assay was conducted in three replicates.

### EAG recording

EAG experiments were conducted to evaluate the antennal responses of adult males, adult females (2-5 day old) and larvae (third instar) of *H. variegata* to volatile compounds identified from cotton aphid-infested plants. For adults, the antennae were carefully excised at the base and tip. Due to small size of the antennae, the head and antennae of larvae were excised. The antennal preparation was immediately placed into glass electrodes filled with electrode solutions containing NaCl (128 mM), CaCl_2_ (1.9 mM), KCl (7.6 mM), and NaHCO_3_ (2.4 mM) (Ockenfels, 2015). The desired concentrations (0.1, 1, 10, and 100 mg/ml) of each compound were prepared using mineral oil as the solvent. Mineral oil and *cis*-3-hexen-1-ol were used as the negative control and reference response, respectively (Xie et al., 2022). To deliver the odorant stimulus, a 20 μl volume of the odor solution was applied onto a Whatman paper strip (1 cm × 3 cm) and inserted into a 1.5 ml micropipette tip. The stimulus was delivered to the antennal sample through a constant flow of clean air (charcoal and humid filtered) at a rate of 300 ml/min for 0.5 s. Each antennal preparation was tested with all compounds in 30-s intervals and replaced with new one for next replicate. For dose-response assay, each antenna was tested against different concentrations of a compound in ascending order. The assay was conducted with 15 replicates. The induced signals were amplified using a 10× AC/DC preamplifier (Syntech, Kirchzarten, Germany), processed with IDAC2 (Syntech, Kirchzarten, Germany), and analyzed using EAGPro V.2 software (Syntech. Kirchzarten, Germany).

### Y-tube behavioral assay

We conducted Y-tube olfactometer trials to determine the behavioral tendencies of adult males, adult females, and third instar larvae of *H. variegata* towards the compounds, identified from infested cotton plants. The olfactometer tubes (diameter: 2.5 cm) were made of glass and consisted of a 20 cm main stem, two 20 cm lateral arms, and a 45° angle between arms. The Y-tube was placed inside a steel chamber (1m × 0.8 m × 0.8 m) which was equipped with a camera and 40-W fluorescent lamps providing uniform lighting (~2000 lux). A video monitor was used to observe the behavioral responses of insects during the assays. A 500 ml glass conical flask served as the odor source container. The lid of each flask contained an air inlet tube connected to a clean air source, and an outlet tube connected to the olfactometer arm. Each compound was individually formulated in light mineral oil to desired concentrations (0.1, 1, 10, and 100 mg/ml), applied as a 20 µl sample on a filter paper strip (50 × 5 mm) and promptly placed inside the treatment flask. Mineral oil alone was used as a control. The stimuli were delivered to the olfactometer arms at a constant air flow rate of 0.3 l/min. Insects were introduced individually at the base of the central arm and given 5 minutes to make a choice. A choice was defined when an insect reached to the midpoint of the lateral arm and remained there for at least 10 seconds. If an insect did not make a choice during this period, it was recorded as no choice and excluded from data analysis. Each insect was used only once. After testing a total of four insects, we switched the position of the treatment and control arms to avoid any positional bias. The Y-tube olfactometer was replaced with a clean one after testing eight individuals. For each odorant, 50 adult males, adult females, or larvae were tested. All olfactometer assays were conducted between 9:00 am to 5:00 pm, under conditions similar to colony maintenance. The entire experiment (testing 50 individuals for each odorant) was repeated 5 times at different days and data are shown as mean ± SEM.

### Statistical analysis

Data analysis were performed using SPSS v25 and Data Analysis Tools package of Microsoft Excel. All data are shown as the mean ± standard error of the mean (SEM). A one-way chi-squared test with the null hypothesis of equal proportion (50: 50) was performed to analyze the preferences of insects between cotton aphid-infested plants and healthy plants in the dual-choice behavioral assay. The relative EAG values were calculated using following formula: EAG relative response= (compound response − control response)/(reference response − control response) (Xie et al., 2022). The data were subjected to ANOVA (one-way or two-way) and Tukey HSD test (*P* < 0.05) to compare the relative EAG responses among adult females, adult males, and larvae, as well as different concentrations of the target compound. For Y-tube behavior assay, a one-way chi-squared test was performed to determine the significant association of choices between two arms (odor source vs. control).

## Results

### Behavioral tendency of *H. variegata* towards infested plant

The behavioral tendency of *H. variegata* individuals in response to healthy and cotton aphid-infested plants was assessed using dual-choice behavioral assays (**Figure 1A**). The results demonstrated a significant attraction of all tested groups including adult females (*x^2^ = *32.496, *P* < 0.001), adult males (*x^2^ = *37.22, *P* < 0.001), and third instar larvae (*x^2^ = *44.24, *P* < 0.001) towards aphid-infested plants. Approximately 65% of tested adult females and males exhibited a preference for the damaged plants. In contrast, approximately 50% of the tested larvae showed no response, which may be attributed to their limited movement abilities (**Figure 1B**).

### Identification of induced volatiles by cotton aphid

**Figure 2:**
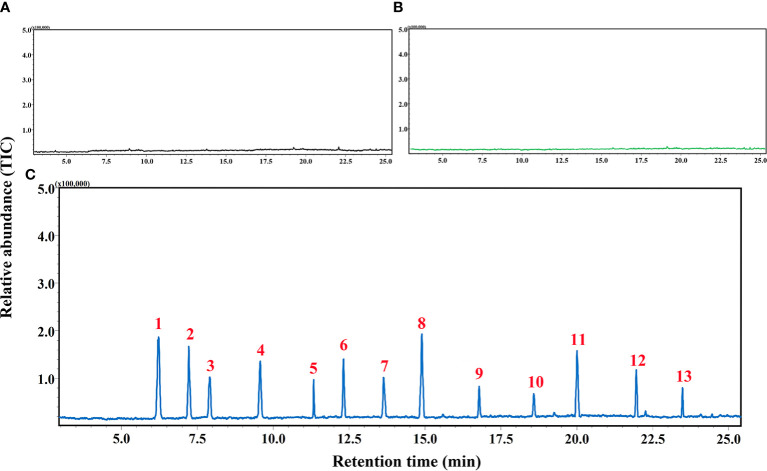
Chromatogram acquired by gas chromatography-mass spectroscopy (GC-MS) analyses of volatile samples collected from **(A)** empty container **(B)** healthy cotton plants, and **(C)** cotton aphid-infested plants. 1) α-Pinene, 2) *cis*-3-Hexenyl acetate, 3) 4-Ethyl-1-octyn-3-ol, 4) β-Ocimene, 5) Dodecane, 6) E-β-Farnesene, 7) Decanal, 8) Methyl salicylate, 9) β-Caryophyllene, 10) α-Humulene, 11) DMNT, 12) Farnesol, 13) TMTT.

GC-MS analyses of samples collected using HS-SPME approach demonstrated no detectable trace of any volatile compounds from negative controls (empty containers) and undamaged cotton plants. In contrast, 13 volatile compounds namely α-pinene, *cis*-3-hexenyl acetate, 4-ethyl-1-octyn-3-ol, β-ocimene, dodecane, E-β-farnesene, decanal, methyl salicylate, β-caryophyllene, α-humulene, (3E)-4,8-dimethyl-1,3,7-nonatriene (DMNT), farnesol, and (E, E)-4,8,12-trimethyltrideca-1,3,7,11-teraene (TMTT) have been detected and identified from the aphid-infested cotton. The concentrations of above-mentioned volatiles induced by the pest were determined by the single point external standard quantification method, which were 25.81, 48.33, 27.88, 62.16, 5.05, 85.73, 77.29, 80.91, 11.07, 4.76, 51.33, 6.72 and 28.69 ng/μl, respectively (**Table 1**; **Figure 2**; **Supplementary Figures S1–14**).

**Table 1:** Retention time and specific information on volatile substances identified from aphid-infested cotton plants.

Number	Volatile substance	Retention time (min)	Structure	Molecular formula	Relative Content (ng/μl)
1	α-Pinene	6.240	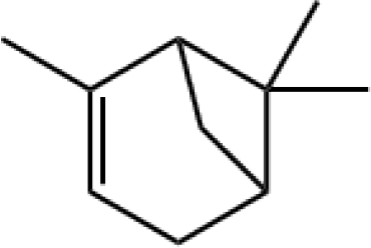	C_10_H_16_	24.39 ± 7.28
2	*cis*-3-Hexenyl acetate	7.245	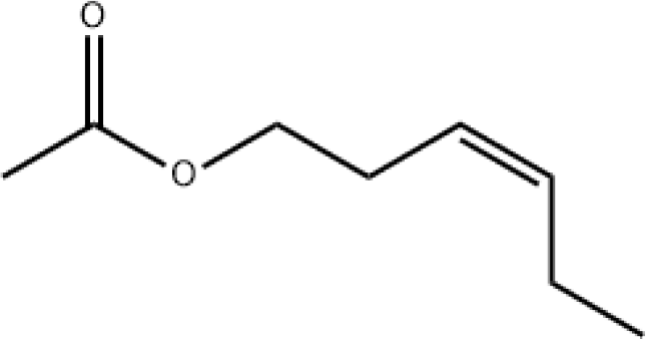	C_8_H_14_O_2_	29.28 ± 8.38
3	4-Ethyl-1-octyn-3-ol	7.925	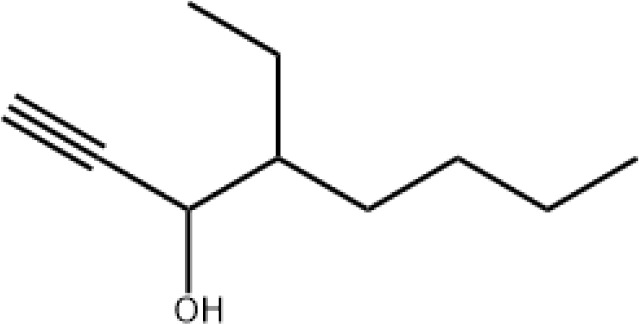	C_10_H_18_O	25.22 ± 9.10
4	β-Ocimene	9.550	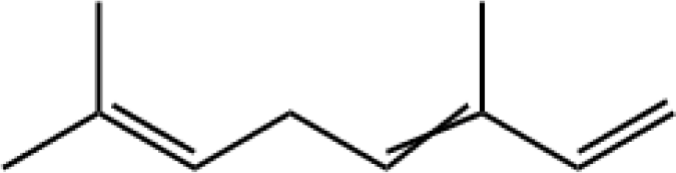	C_10_H_16_	62.61 ± 25.74
5	Dodecane	11.265	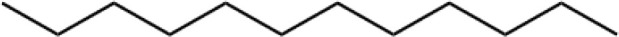	C_12_H_26_	6.43 ± 0.92
6	E-β-Farnesene	12.295	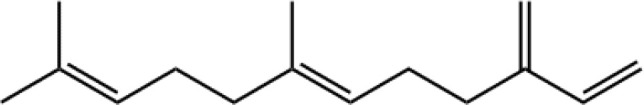	C_15_H_24_	57.53 ± 11.60
7	Decanal	13.675	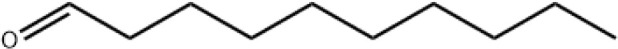	C_10_H_20_O	83.79 ± 27.41
8	Methyl salicylate	14.975	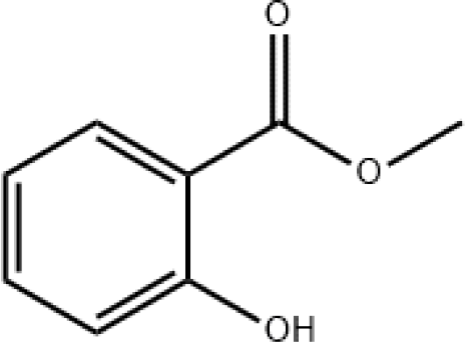	C_8_H_8_O_3_	89.40 ± 11.03
9	β-Caryophyllene	16.765	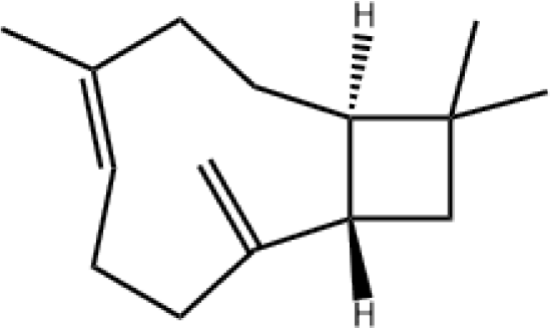	C_15_H_24_	8.02 ± 1.78
10	α-Humulene	18.515	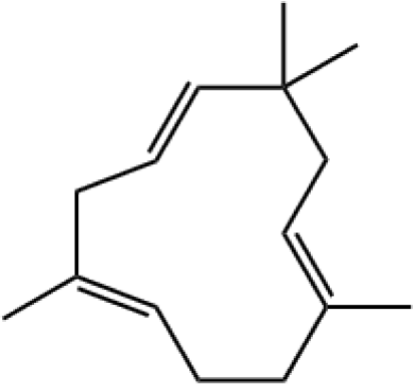	C_15_H_24_	5.52 ± 0.52
11	DMNT	20.005	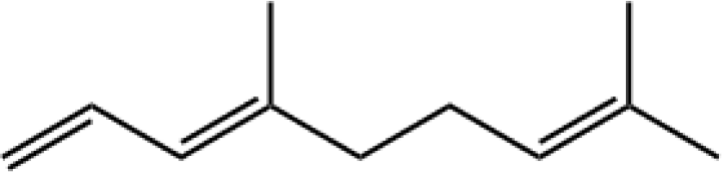	C_11_H_18_	51.97 ± 17.88
12	Farnesol	21.975	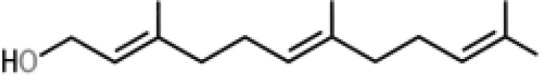	C_15_H_26_O	7.99 ± 1.35
13	TMTT	23.495	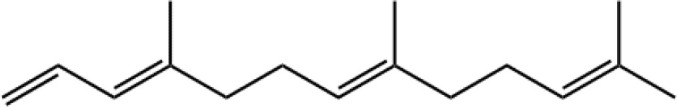	C_16_H_26_	28.36 ± 5.27

### Electrophysiological responses of *H. variegata* antennae

**Figure 3:**
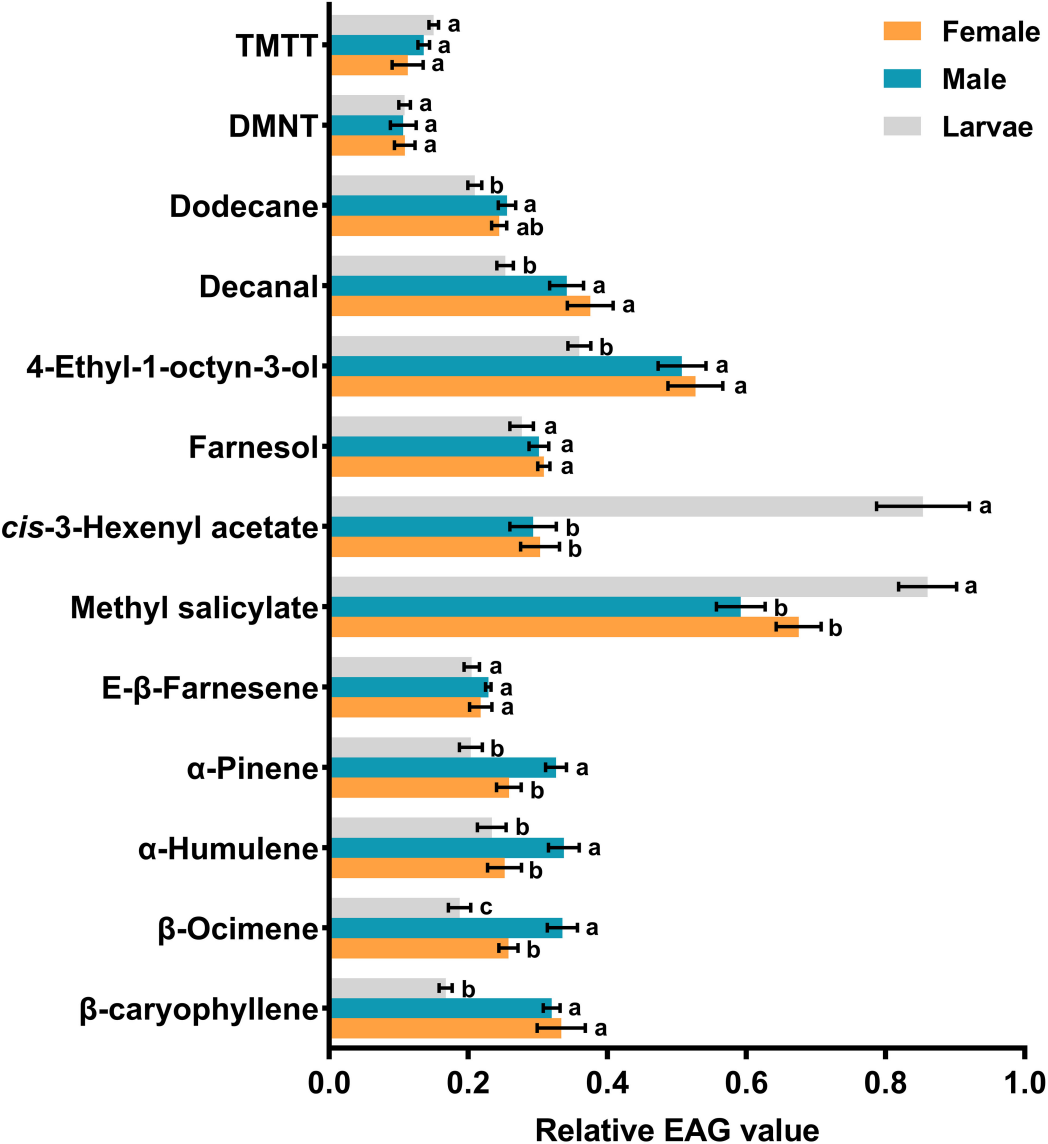
Electroantennogram (EAG) responses of third instar larvae, adult males and adult females of *Hippodamia variegata* to cotton aphid induced volatiles at the concentrations of 10 mg/ml. The data are shown as mean ± SEM. For each chemical, different letters show significant differences, analyzed by one-way ANOVA and Tukey HSD test (*P* < 0.05).

**Figure 4:**
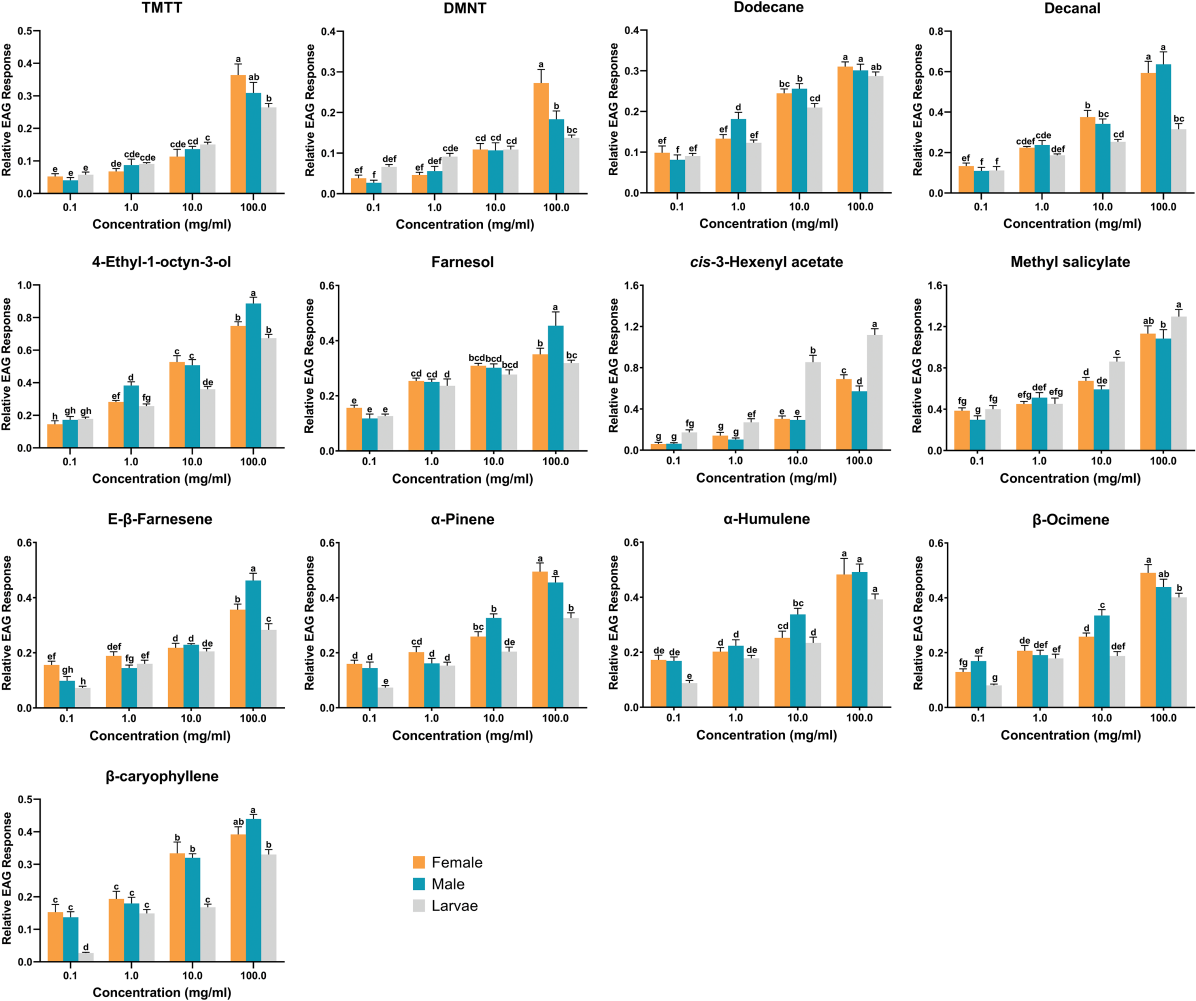
The dose-dependent electroantennogram (EAG) responses of third instar larvae, adult males and adult females of *Hippodamia variegata* to cotton aphid induced volatiles. The data are shown as mean ± SEM. For each chemical, different letters show significant differences, analyzed by two-way ANOVA and Tukey HSD test (*P* < 0.05).

The results of EAG assays revealed that all 13 identified compounds were electrophysiologically active and elicited antennal response in adult females, adult males and third instar larvae. Among the tested compounds, methyl salicylate (*df* = 2, *f* = 9.762, *P* = 0.001) and *cis*-3-hexenyl acetate (*df* = 2, *f* = 45.12, *P* < 0.001) triggered significantly larger responses in larval antennae compared to male and female antennae at the concentration of 10 mg/ml. Conversely, β-ocimene (*df* = 2, *f* = 12.285, *P* < 0.001), α-humulene (*df* = 2, *f* = 4.835, *P* = 0.019), and α-pinene (*df* = 2, *f* = 10.165, *P* = 0.001) elicited significantly greater responses in male antennae. On the other hand, no significant differences were observed among antennal responses of females, males, and larvae when exposed to TMTT, DMNT, E-β-farnesene, and farnesol (**Figure 3**). In addition, dose-dependent EAG recording indicated that all tested compounds exhibited increasing responses in a concentration-dependent manner, from the lowest concentration (0.1 mg/ml) to the highest tested concentration (100 mg/ml). While no significant differences were recorded among larvae, adult males and adult females in response to certain chemicals at 10 mg/ml, however, increasing the concentrations to 100 mg/ml induced distinct variations. For example, TMTT and DMNT elicited significantly higher EAG responses in adult females compared to adult males and larvae. Similarly, 4-ethyl-1-octyn-3-ol, E-β-farnesene, and farnesol triggered significantly higher EAG responses in adult males compared to adult females and larvae (**Figure 4**).

### Behavioral responses of *H. variegata* to aphid-induced volatiles

**Figure 5:**
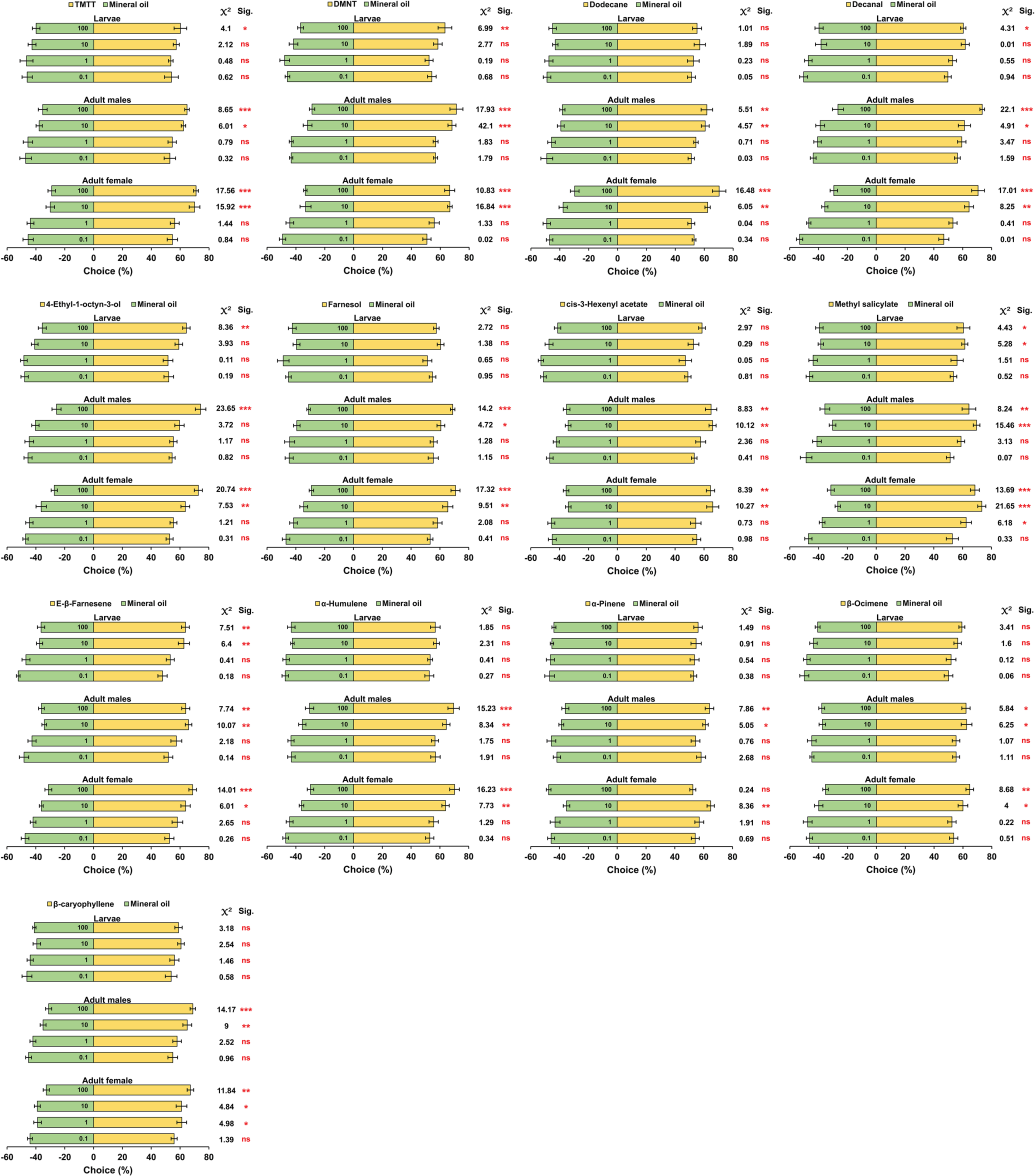
The dose-dependent behavioral responses of third instar larvae, adult males and adult females of *Hippodamia variegata* to cotton aphid induced volatiles in the Y-tube olfactometer test. The data are shown as mean ± SEM. “ns”, “*”, “**”, and “***” indicate no significant difference, significant difference at the *P*< 0.05 level, significant difference at the *P*< 0.01 level, and significant difference at the *P*< 0.0001 level, respectively, analyzed by chi-squared test.

The results of Y-tube olfactometer trials demonstrated that at the lowest concentration (0.1 mg/ml), adult females, adult males and larvae had no preferences to all tested chemicals. Similarly, at 1 mg/ml, no preferences were observed in all the treatments except for β-caryophyllene (*x^2^* = 4.98, *P* = 0.0255) and methyl salicylate (*x^2^* = 6.18, *P* = 0.0128), which were found to be attractive only to adult females. Increasing the concentration to 10 and 100 mg/ml significantly influenced behavioral tendencies of tested individuals, and in most treatments, females, males, and larvae exhibited obvious preferences for the aphid-induced volatiles over the mineral oil. However, the third instar larvae exhibited no preferences for dodecane (*x^2^* = 1.016, *P* = 0.314), farnesol (*x^2^* = 2.275, *P* = 0.131), *cis*-3-hexenyl acetate (*x^2^* = 2.972, *P* = 0.084), α-humulene (*x^2^* = 1.583, *P* = 0.173), α-pinene (*x^2^* = 1.493, *P* = 0.221), β-ocimene (*x^2^* = 3.414, *P* = 0.065), and β-caryophyllene (*x^2^* = 3.181, *P* = 0.074) even at highest concentration (100 mg/ml) (**Figure 5**).

## Discussion

In this study, we comprehensively studied the ecological function of aphid-induced volatiles emitted by cotton plants on an important natural enemy. Through behavioral assay, we have demonstrated that both the immature stage and adult stages of *H. variegata* exhibited a clear preference for damaged cotton plants over healthy ones. This phenomenon was reported in several previous studies. For instance, sweet pepper plants injured by *A. gossypii* and *Myzus persicae* were found to be attractive to the ladybird predator *Cycloneda sanguinea*, while uninjured plants did not elicit the same response (Oliveira and Pareja, 2014). Investigations on the behavioral tendencies of ladybirds such as *Coccinella septempunctata* and *Harmonia axyridis* towards aphid infested and uninfested plants consistently showed that the ladybirds preferentially selected the infested ones (Rondoni et al., 2017; Xiu et al., 2019; Norkute et al., 2020). As mentioned earlier, the emission of induced volatiles upon the aphid infestation triggers such behaviors in a variety of natural enemies, including ladybirds.

The specific blend of HIPVs emitted by a plant depends on multiple factors such as plant growth conditions, plant and herbivore species, as well as plant and herbivore developmental stage. Conversely, different methodological approaches result in the collection and identification of various HIPVs from the interaction between the same plant and herbivore (Heil, 2008; Oliveira and Pareja, 2014). In a previous study, only four HIPVs, including DMNT, TMTT, methyl salicylate, and (*cis*)-3-hexenyl acetate, were reported from cotton plants infested with cotton aphids (Hegde et al., 2011). However, in this study, we identified a greater number of compounds from cotton plant damaged by *A. gossypii*. In total, 13 compounds from different group of chemicals such as terpenes, esters, aliphatic alcohol, alkane, and aldehyde were emitted from aphid-infested plants. In our study, the majority (8 out of 13) of emitted volatiles induced by *A. gossypii* were found to be from different subclasses of terpenes including monoterpenes (α-pinene and β-ocimene), sesquiterpenes (E-β-farnesene, β-caryophyllene, α-humulene, and farnesol), and homoterpenes (DMNT and TMTT). These results are in good agreements with previous reports (McCall et al., 1994; Hegde et al., 2011; Arce et al., 2021). Terpenoids are a large and diverse class of organic compounds that are locally or systemically produced by plants in response to herbivores. They play a variety of ecological roles, including attracting natural enemies such as ladybirds and repelling herbivores (Cheng et al., 2007; Mumm et al., 2008; Xu et al., 2023).

EAG experiments revealed the electrophysiological activity of all identified compounds, eliciting noticeable responses in third instar larvae and adults of *H. variegata*. However, despite these responses, the third instar larvae displayed no preferences towards several compounds. For instance, while *cis*-3-hexenyl acetate elicited antennal responses in larvae at all tested concentrations, with significantly larger responses compared to adult males and females in EAG tests, no distinct preferences were observed in Y-tube choice assays. This inconsistency appears prevalent in insect olfactory behavior, where responses in EAG and behavioral bioassays do not consistently align (Yan et al., 2018; Khashaveh et al., 2020). Yet, answering this puzzling observation remains challenging within current knowledge boundaries. One possible explanation for this discrepancy lies in the inherent limitations of EAG recordings, which primarily assess the activity of olfactory receptors in the antennae. Conversely, behavioral responses encompass a broader spectrum of physiological and cognitive processes, integrating sensory information from multiple modalities. Moreover, behavioral effects may arise from synergistic or antagonistic interactions between individual components. Studying odorants in isolation might yield an incomplete understanding of their role in insect behavior. Additionally, it’s important to consider that most olfactometer studies focus on short-range orientation behaviors, while some chemical cues may function at long distances and trigger more complex behaviors (Clifford and Riffell, 2013; Webster and Carde, 2017; Shao et al., 2021; Saha, 2022).

In Y-tube behavioral trials, it was evident that, apart from those compounds that were inactive for larvae, all the tested compounds were behaviorally attractive to both the larvae and adults of *H. variegata*, particularly at the highest applied concentrations (10 and 100 mg/ml). Behavioral assays of insects in laboratory conditions may require higher concentrations of volatile compounds due to several factors. Laboratory assays often employ standardized odor stimuli, presented in a controlled manner that may deviate from the natural spatiotemporal patterns of odor cues in the environment. In nature, insects are accustomed to encountering odor plumes that vary in intensity and duration, creating a dynamic olfactory landscape. However, in laboratory assays, odor stimuli are often presented at constant concentrations and durations, potentially reducing the responsiveness of insects. In addition, laboratory-reared insects may exhibit reduced olfactory sensitivity compared to their wild counterparts due to the lack of exposure to natural olfactory stimuli and the potential for genetic drift in laboratory populations. This reduced sensitivity may necessitate the use of higher concentrations of volatile compounds to elicit a behavioral response (Silva et al., 2019; Reddy et al., 2022).

Several research works have reported the effects of HIPVs on *H. variegata* and other coccinellid beetles (Ninkovic et al., 2001; Gençer et al., 2017; Xiu et al., 2019; Norkute et al., 2020; Xie et al., 2022; Jiang et al., 2023; Xu et al., 2023). Methyl salicylate was found to attract *H. variegata* adults in Y-tube olfactometer, however a notably higher level of attraction was observed when this compound was tested in combination with benzaldehyde or farnesene in a binary setup (Gençer et al., 2017). In laboratory conditions, several aphid-induced volatiles including α-pinene from *Glycyrrhiza uralensis* and *Alhagi sparsifolia* (Fabales: Fabaceae) were shown to be attractive to *H. variegata* adults (Jiang et al., 2023). In our previous study, we showed that aphid-induced volatile *cis*-3-hexenyl acetate were attractive to males and females of *H. variegata* (Xie et al., 2022). Cotton plants, when subjected to herbivore feeding or damage, can release a range of volatile organic compounds, including the sesquiterpene E-β-farnesene (Rose et al., 1996; Rose and Tumlinson, 2004). Intriguingly, this compound is the major compound of alarm pheromone in various aphid species (Pickett and Griffiths, 1980). Studies demonstrated that E-β-farnesene are highly attractive to larvae, adult males and adult females of ladybirds such as *H. axyridis* and *Adalia bipunctata* (Francis et al., 2004; Verheggen et al., 2007). These reports, which are in good agreements with outcomes of our study, emphasize that researches on HIPVs contribute immensely to understanding plant-insect interactions.

In conclusion, the identified active compounds emitted from aphid-infested cotton plants were proven to be attractive to *H. variegata*. Our previous study demonstrated that synthetic HIPVs significantly influenced the attraction of ladybirds in cotton fields (Yu et al., 2018). However, further filed studies are recommended to evaluated the practical application of these behaviorally active compounds. Utilizing these active volatiles in various types of traps may introduce the ladybirds into the cotton fields earlier and help to stay over a longer time. These facilities the temporal and spatial overlap between natural enemies and pests, thereby enhancing the effectiveness of biological control.

## Data availability statement

The original contributions presented in the study are included in the article/**Supplementary Material**. Further inquiries can be directed to the corresponding authors.

## Ethics statement

The animal study was approved by Experimental Animal Welfare and Ethical Committee of Institute of Plant Protection, Chinese Academy of Agricultural Sciences. The study was conducted in accordance with the local legislation and institutional requirements. No potentially identifiable images or data are presented in this study.

## Author contributions

CY: Data curation, Investigation, Methodology, Visualization, Writing – original draft, Writing – review & editing. DT: Data curation, Formal analysis, Investigation, Methodology, Visualization, Writing – review & editing. JX: Investigation, Methodology, Writing – review & editing. HT: Investigation, Methodology, Writing – review & editing. DZ: Investigation, Methodology, Writing – review & editing. XL: Formal analysis, Software, Visualization, Writing – review & editing. TL: Supervision, Validation, Writing – review & editing. WD: Supervision, Validation, Writing – review & editing. AK: Conceptualization, Funding acquisition, Writing – original draft, Writing – review & editing. YZ: Conceptualization, Funding acquisition, Supervision, Validation, Writing – original draft, Writing – review & editing.
