# Supplementary material for: Volatiles from cotton aphid (Aphis gossypii) infested plants attract the natural enemy Hippodamia variegata
Source: Front Plant Sci. 2023 Dec 20;14:1326630. doi: 10.3389/fpls.2023.1326630 (PMC10761428; doi:10.3389/fpls.2023.1326630)
Supplement: Supplementary file 1 [file DataSheet_1.docx]

**Table S1. Volatile substances used in EAG and Y-tube assays**

| Number | Volatile substance | Source |  | CAS number | Purity |
| --- | --- | --- | --- | --- | --- |
| 1 | α-Pinene | TCI |  | 80-56-8 | >98.0 |
| 2 | cis-3-Hexenyl acetate | TCI |  | 3681-71-8 | >98.0 |
| 3 | 4-Ethyl-1-octyn-3-ol | TCI |  | 5877-42-9 | >97.0 |
| 4 | β-Ocimene | TCI |  | 13877-91-3 | ≥90.0 |
| 5 | Dodecane | TCI |  | 112-40-3 | >98.0 |
| 6 | *E*-β-Farnesene | TCI |  | 18794-84-8 | ≥90.0 |
| 7 | Decanal | TCI |  | 112-31-2 | >97.0 |
| 8 | Methyl salicylate | TCI |  | 119-36-8 | >98.0 |
| 9 | β-caryophyllene | TCI |  | 87-44-5 | >98.0 |
| 10 | α-Humulene | TCI |  | 6753-98-6 | >93.0 |
| 11 | DMNT | Chemical book |  | 95452-08-7 | >95.0 |
| 12 | Farnesol | TCI |  | 4602-84-0 | ≥95.0 |
| 13 | TMTT | Chemical book |  | 62235-06-7 | >95.0 |
| 14 | cis-Hex-3-en-1-ol | Sigma |  | 928-96-1 | ≥98.0 |
| 15 | Mineral oil | Aladdin |  | 8042-47-5 | ultra-pure |
| 16 | n-Hexane | Sigma |  | 110-54-3 | 99% |

**Figure S1**. Linear relationship between peak area and concentration of α-pinene


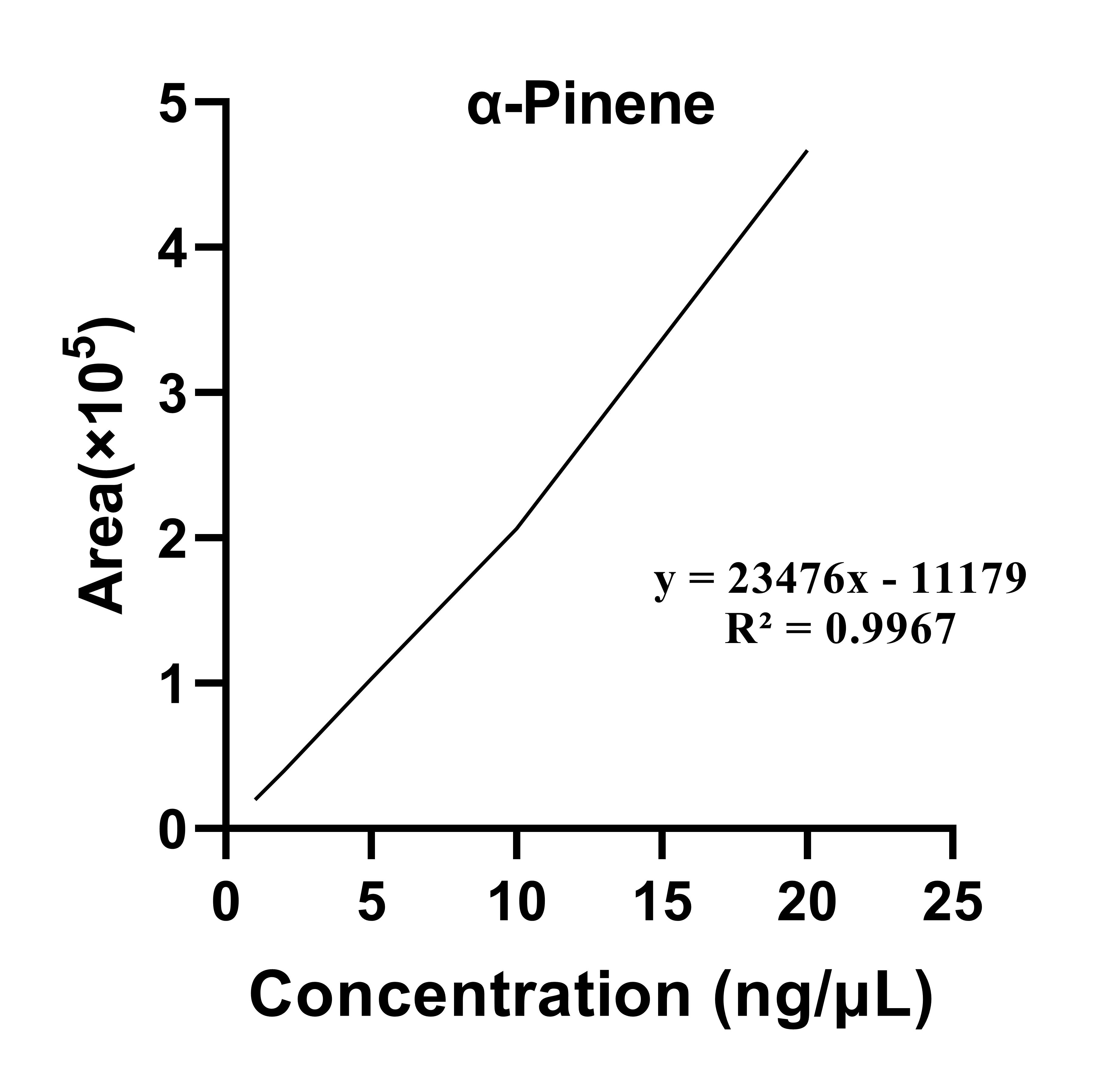


**Figure S2**. Linear relationship between peak area and concentration of *cis*-3-hexenyl acetate


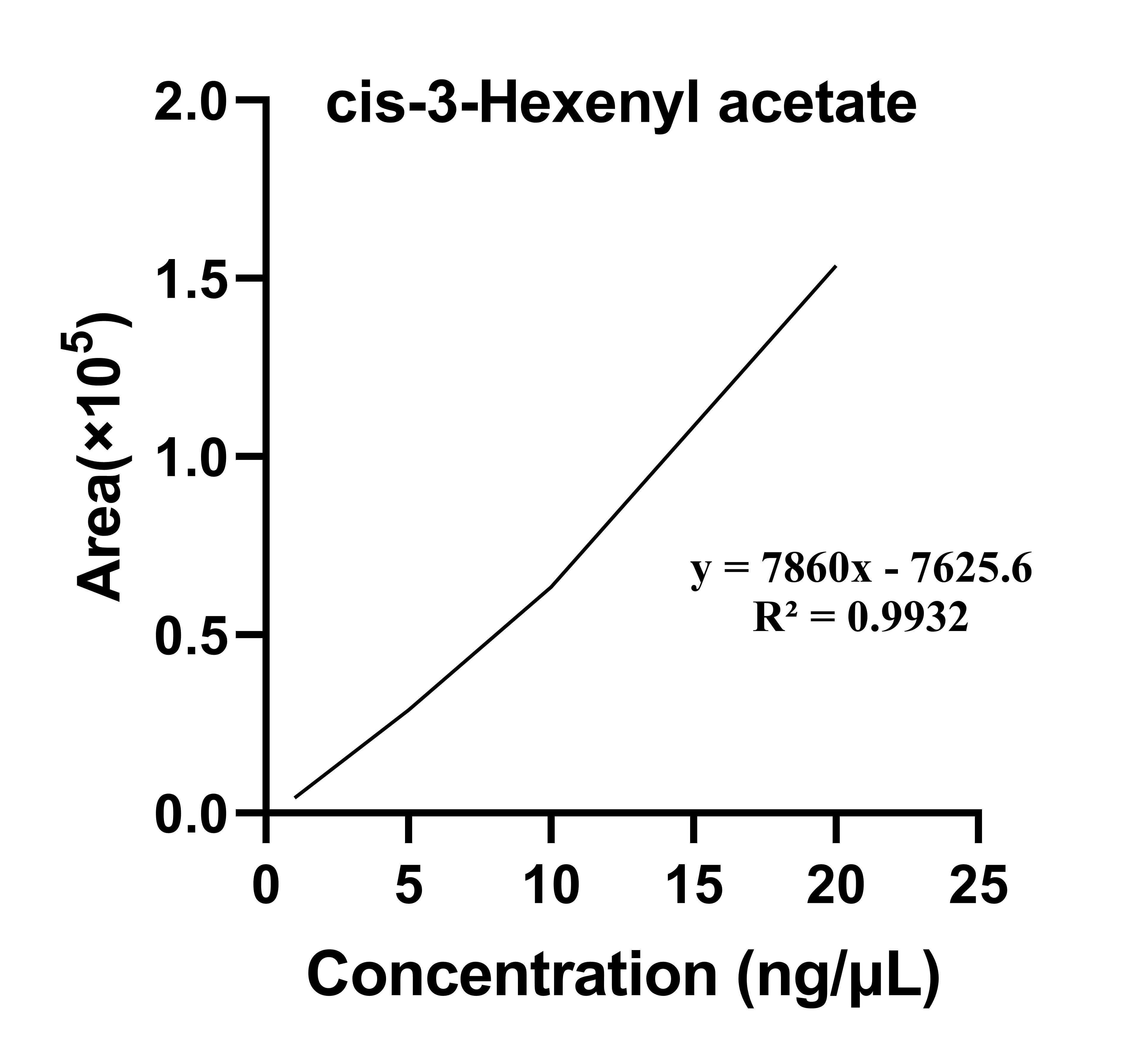


**Figure S3**. Linear relationship between peak area and concentration of 4-ethyl-1-octyn-3-ol


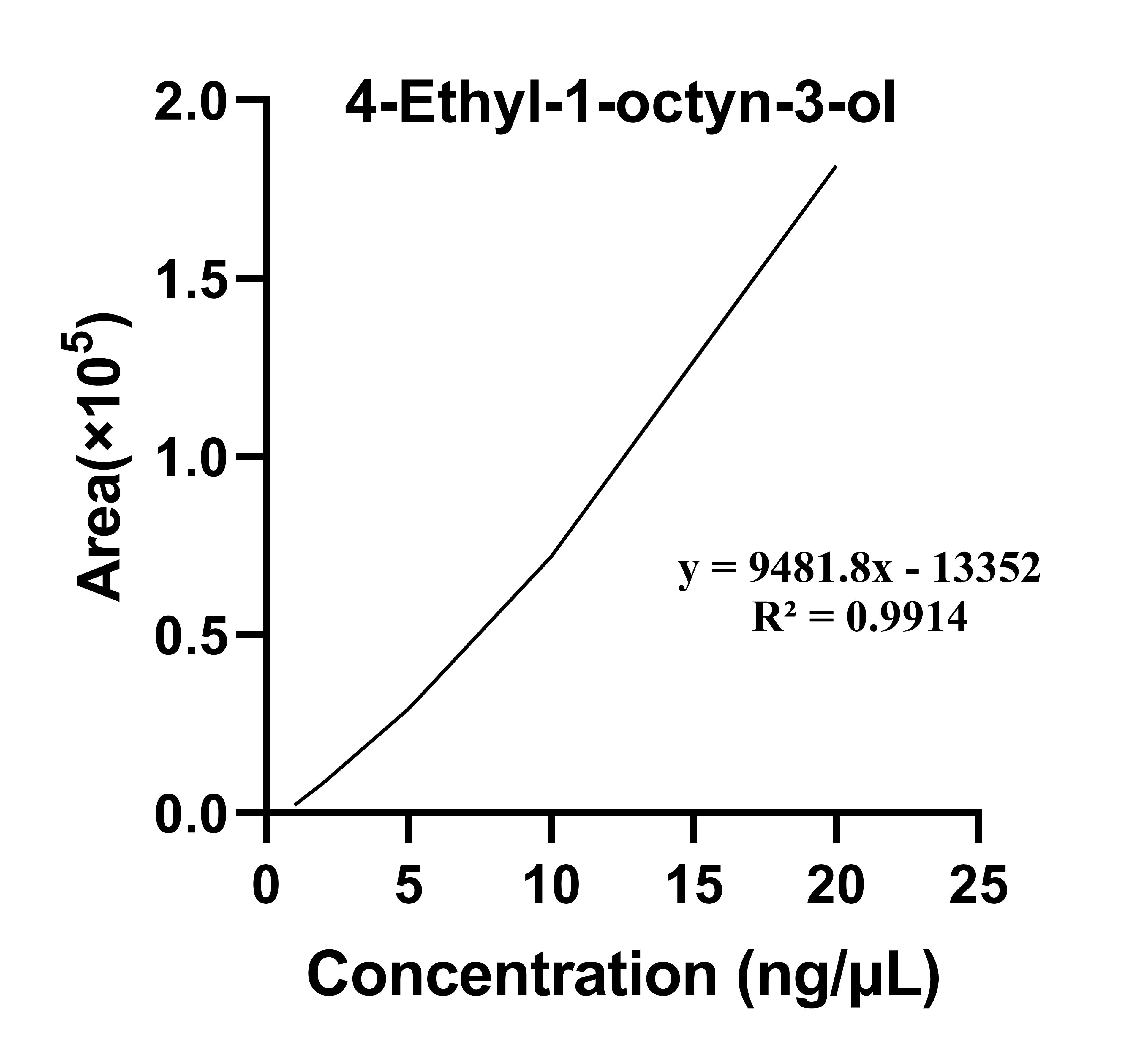


**Figure S4**. Linear relationship between peak area and concentration of β-ocimene


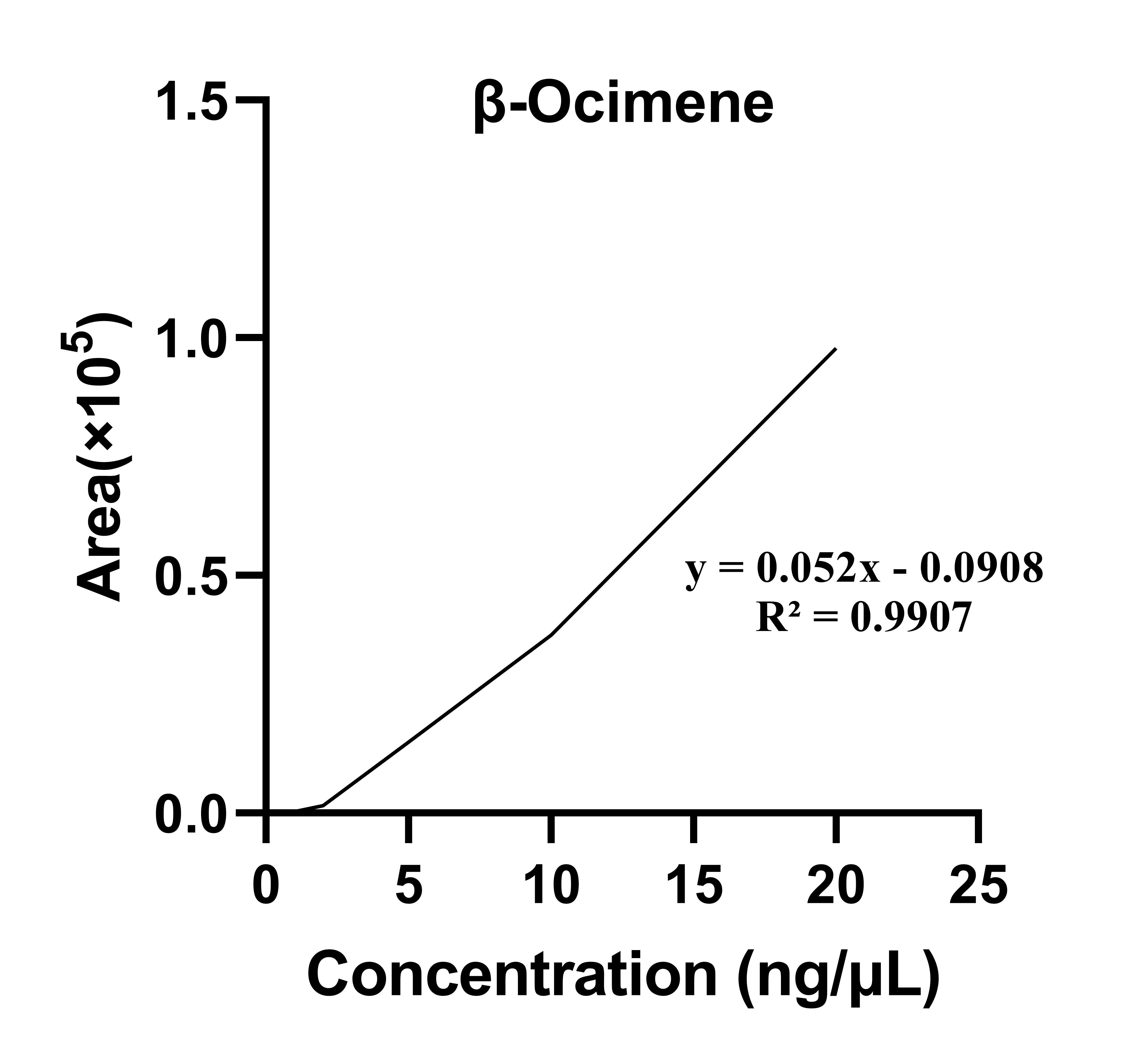


**Figure S5**. Linear relationship between peak area and concentration of dodecane


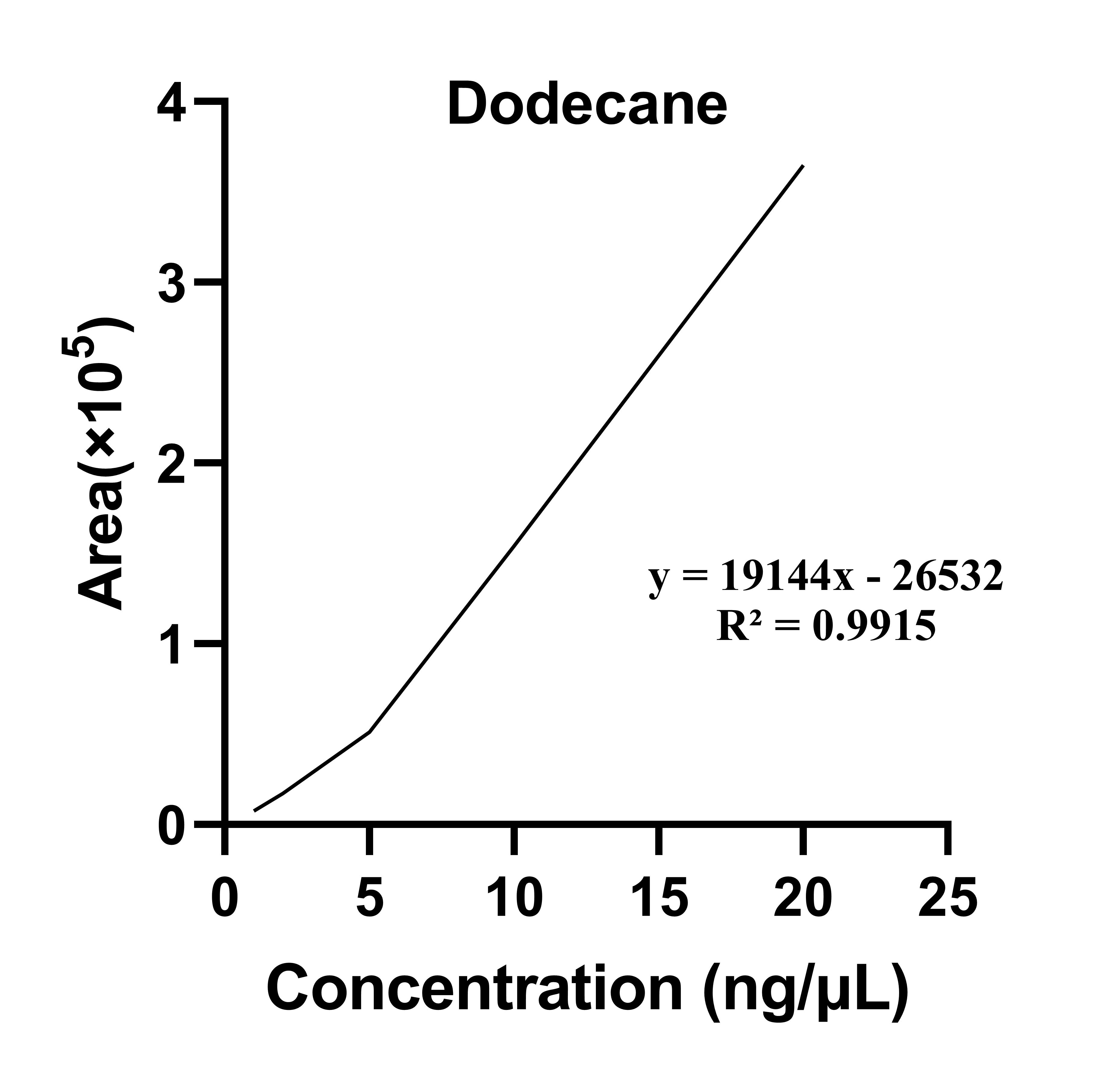


**Figure S6**. Linear relationship between peak area and concentration of E-β-farnesene


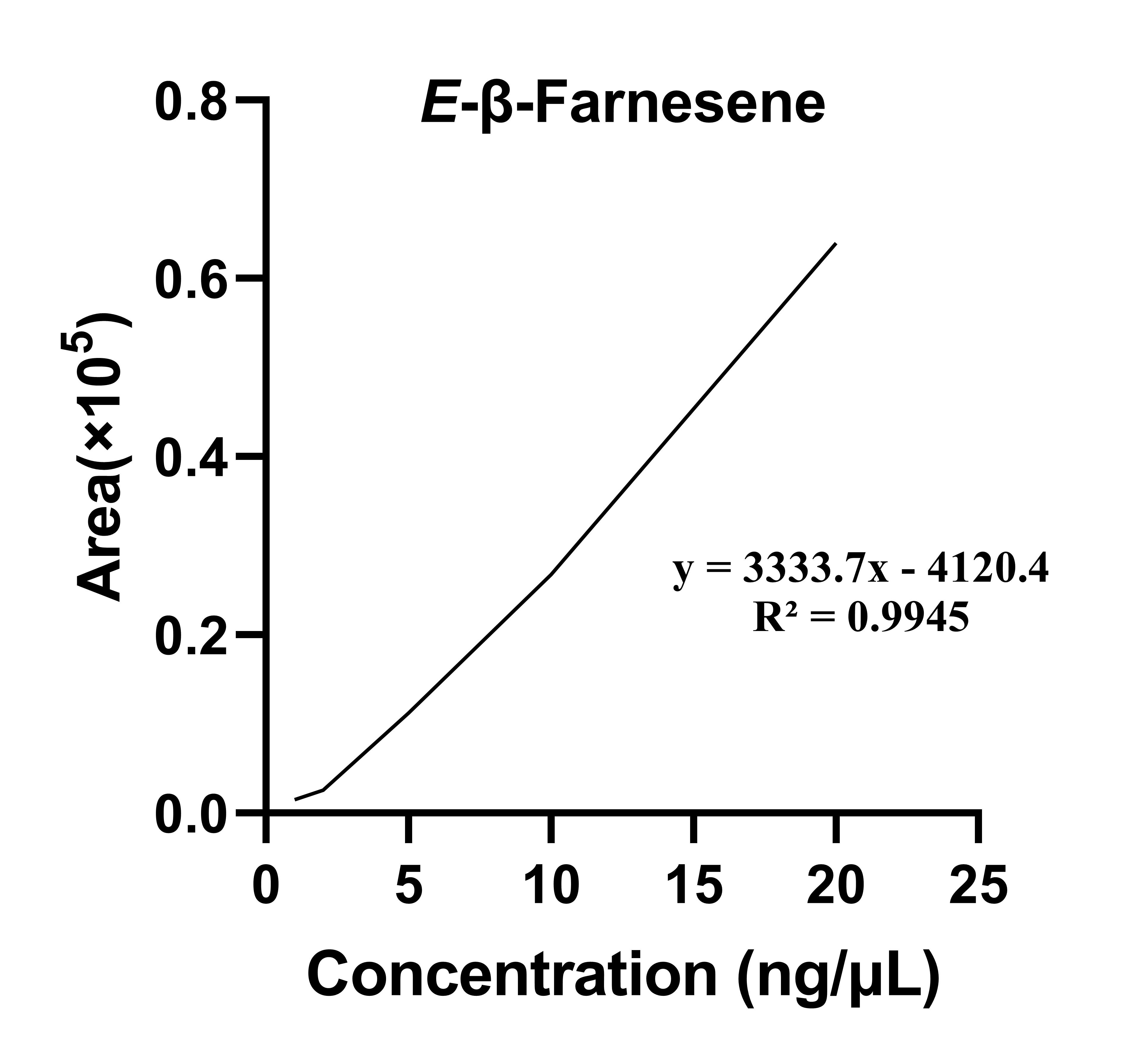


**Figure S7**. Linear relationship between peak area and concentration of decanal


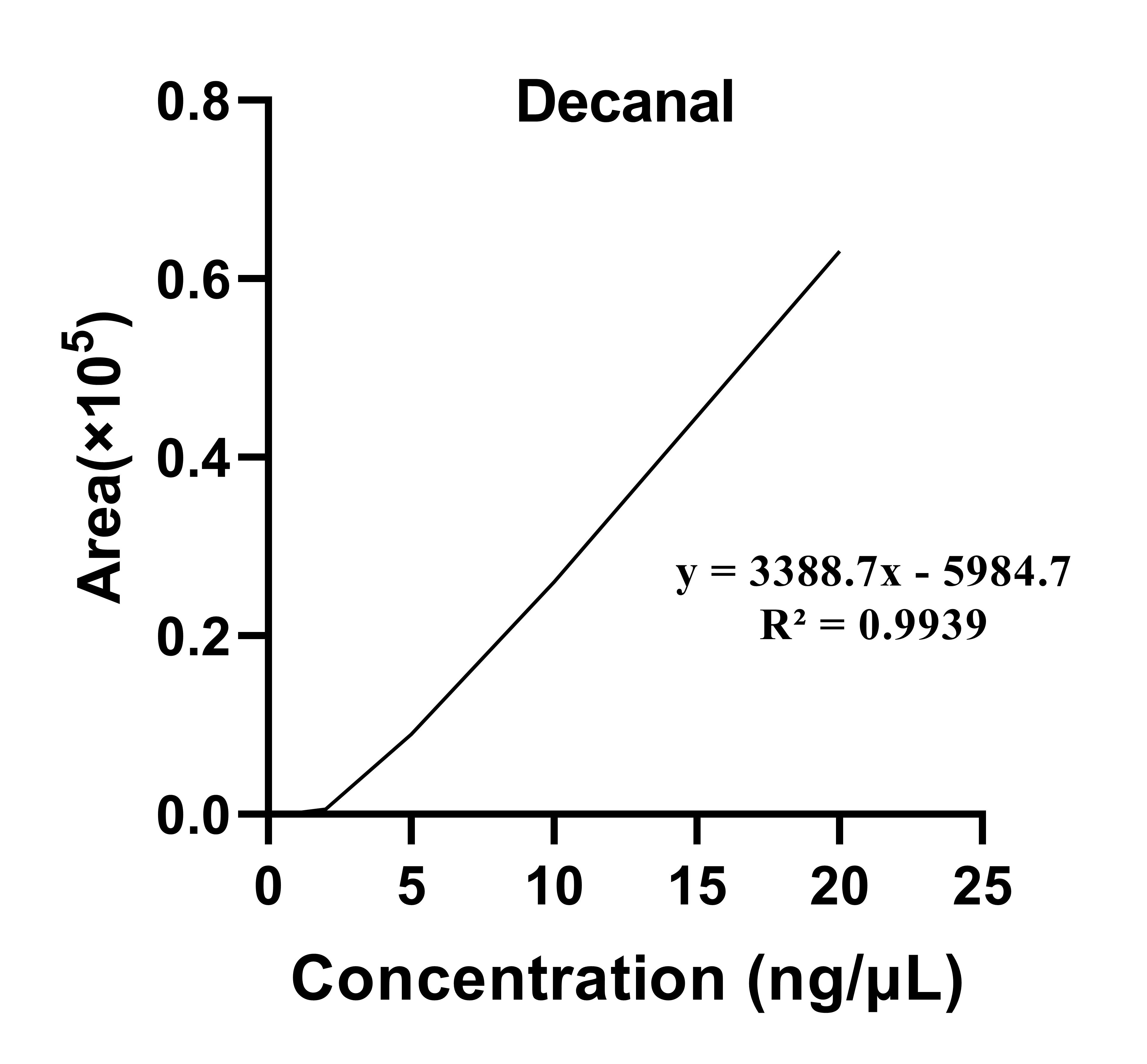


**Figure S8**. Linear relationship between peak area and concentration of methyl salicylate


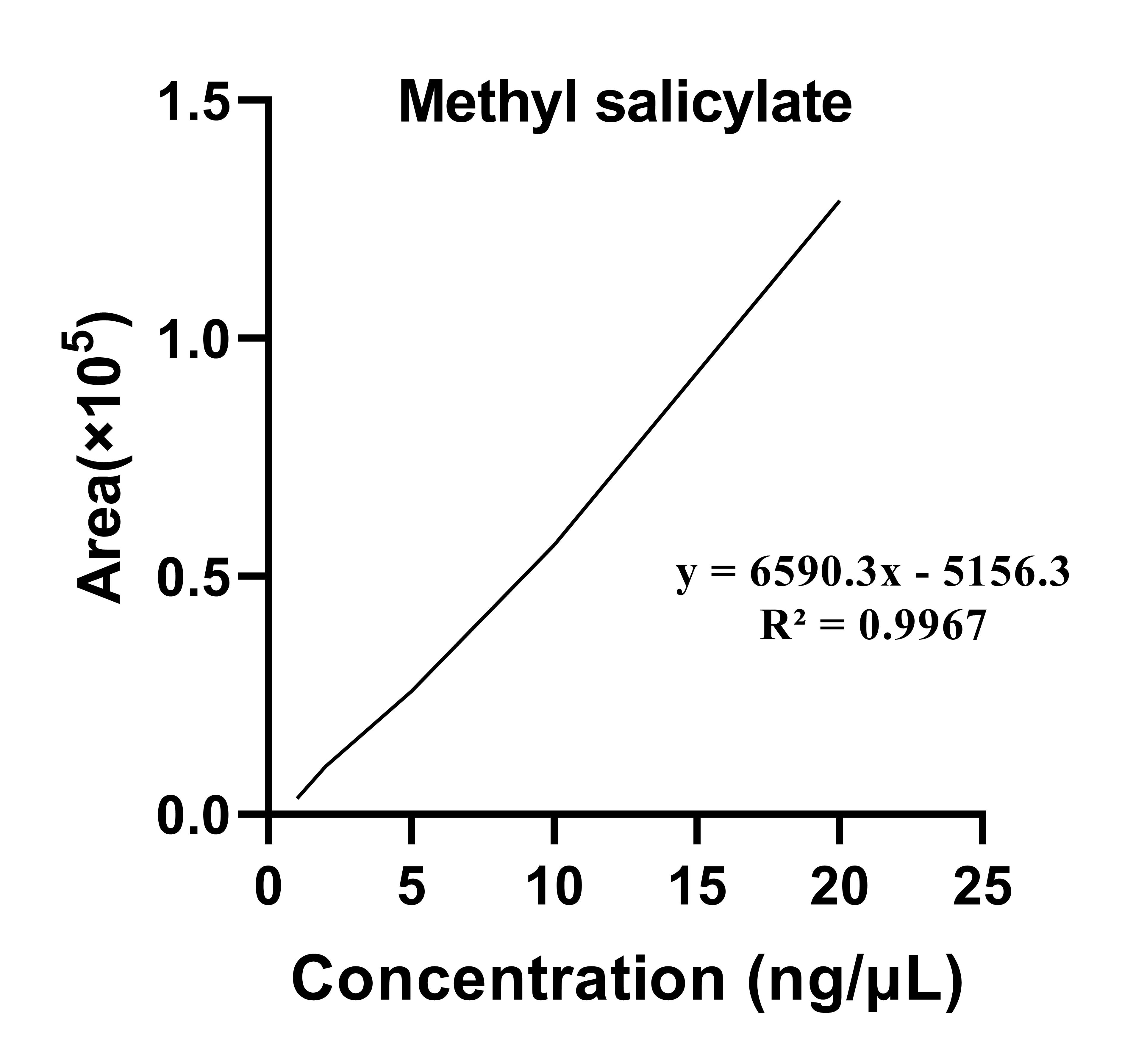


**Figure S9**. Linear relationship between peak area and concentration of β-caryophyllene


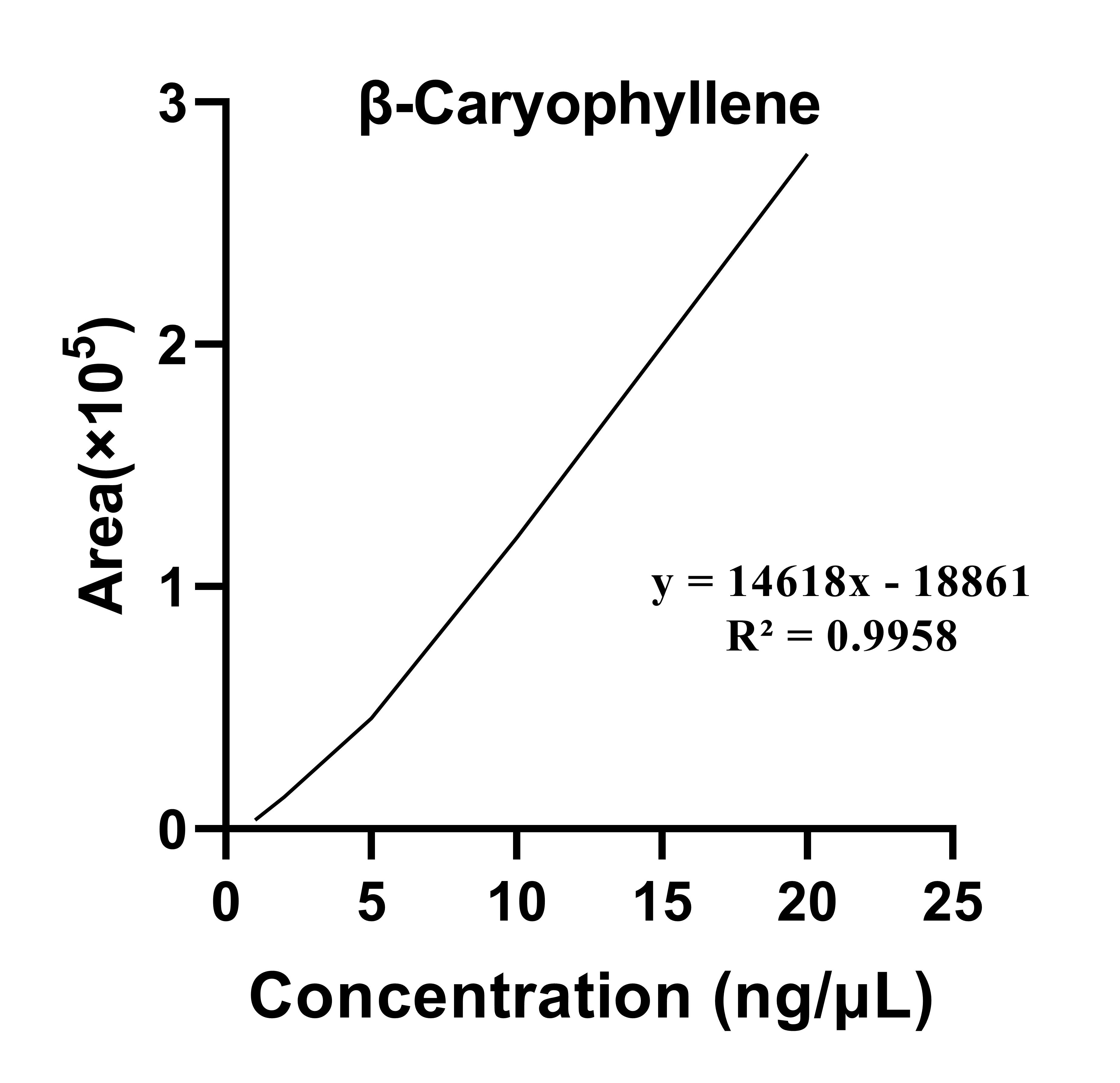


**Figure S10**. Linear relationship between peak area and concentration of α-humulene


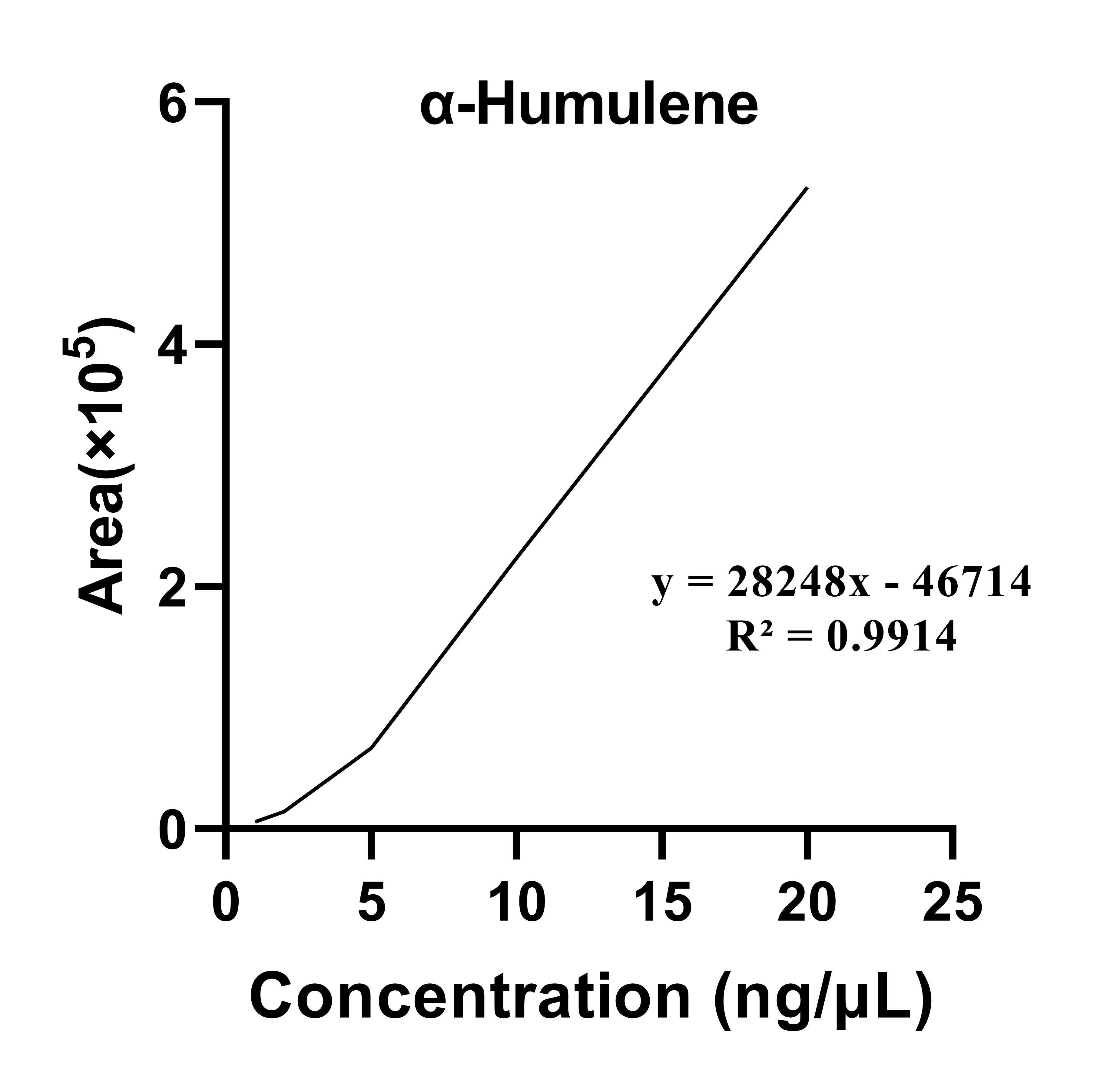


**Figure S11**. Linear relationship between peak area and concentration of DMNT


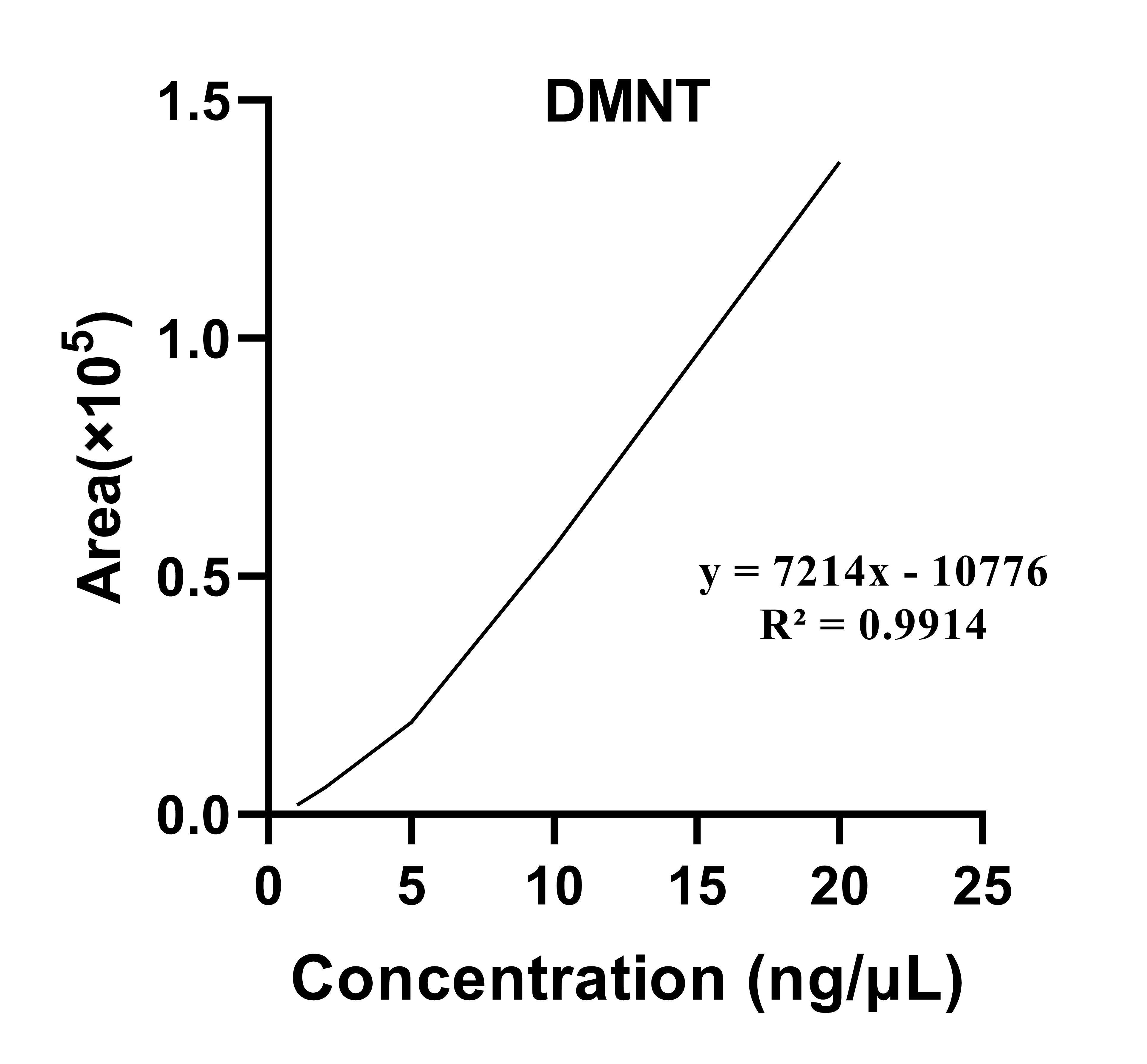


**Figure S12**. Linear relationship between peak area and concentration of farnesol


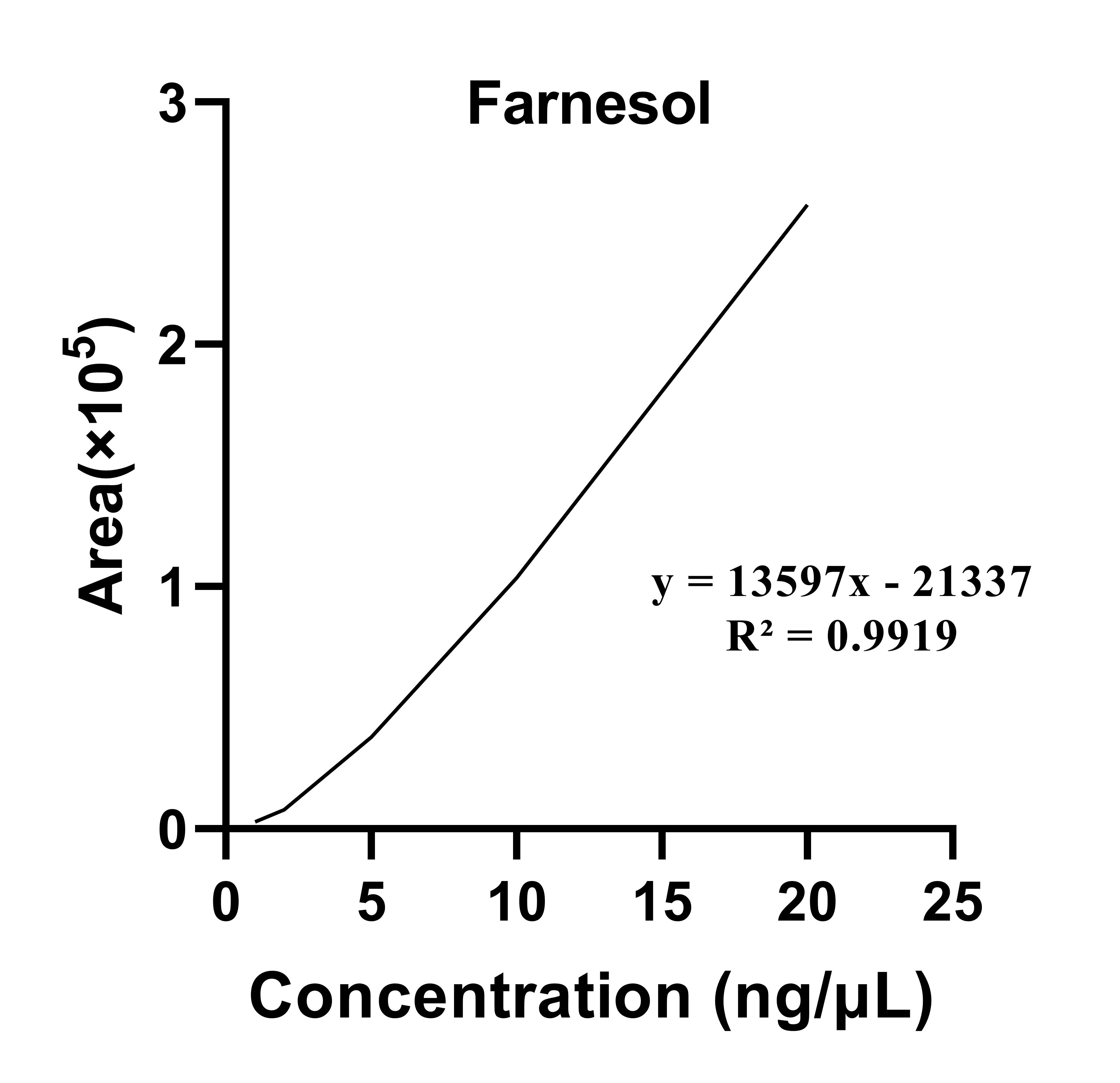


**Figure S13**. Linear relationship between peak area and concentration of TMTT


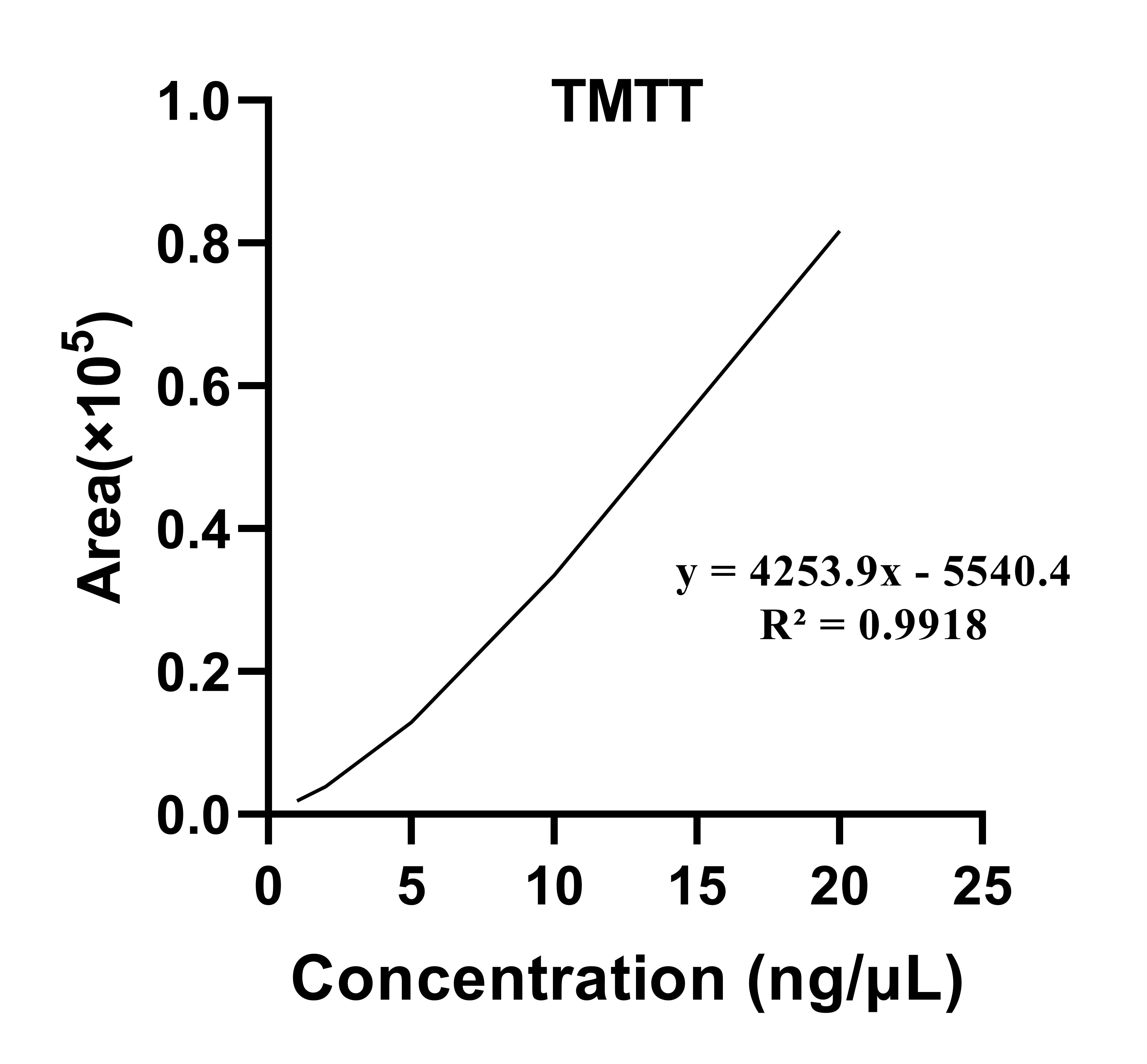


**Figure S14.** The chromatogram of the standard compound solutions in n-hexane
